# Supplementary material for: Prevalence and correlates of prescription opioid use among US adults, 2019–2020
Source: PLoS One. 2023 Mar 2;18(3):e0282536. doi: 10.1371/journal.pone.0282536 (PMC9980762; doi:10.1371/journal.pone.0282536)
Supplement: S1 File — (DOCX) [file pone.0282536.s001.docx]

**SUPPLEMENTAL TABLES**

**Prevalence and Correlates of Prescription Opioid Use among US Adults in 2019-2020.**

| Supplemental Table S1. Characteristics of the target population, US adults 18 and older, NHIS 2019/2020 | | | |
| --- | --- | --- | --- |
|  | Percent | Number valid | Number and percent missing |
| Survey year |  | 53,150 | 0 |
| 2019 | 49.9 |  |  |
| 2020 | 50.1 |  |  |
| Region of residence |  | 53,150 | 0 |
| Northeast | 17.7 |  |  |
| North Central/Midwest | 21.0 |  |  |
| South | 37.8 |  |  |
| West | 23.6 |  |  |
| Rural/urban county classification |  | 53,150 | 0 |
| Large central metro | 30.4 |  |  |
| Large fringe metro | 25.0 |  |  |
| Medium and small metro | 30.5 |  |  |
| Nonmetropolitan | 14.1 |  |  |
| Age |  | 53,018 | 132 (0.2%) |
| 18-44 | 45.9 |  |  |
| 45-64 | 32.8 |  |  |
| 65+ | 21.4 |  |  |
| Female |  | 53,146 | 4 (<0.0%) |
| Male | 48.3 |  |  |
| Female | 51.7 |  |  |
| Race/ethnicity |  | 53,150 | 0 |
| White | 63.1 |  |  |
| Black | 11.7 |  |  |
| Hispanic | 16.6 |  |  |
| Other | 8.5 |  |  |
| Immigrant status |  | 51,679 | 1,471 (2.8%) |
| US-born | 81.7 |  |  |
| Foreign-born | 18.3 |  |  |
| Marital status |  | 51,596 | 1,554 (2.9%) |
| Married | 52.5 |  |  |
| Previously married | 18.7 |  |  |
| Never married | 28.9 |  |  |
| Educational attainment |  | 52,861 | 289 (0.5%) |
| Less than high school | 12.1 |  |  |
| GED | 2.4 |  |  |
| High school diploma | 25.2 |  |  |
| Some college, no diploma | 17.6 |  |  |
| Associate degree | 13.3 |  |  |
| Bachelor’s degree | 18.5 |  |  |
| Master’s or higher degree | 10.9 |  |  |
| Family income |  | 53,150 | 0 |
| $0-34,999 | 24.1 |  |  |
| $35,000-49,999 | 12.9 |  |  |
| $50,000 - $74,999 | 18.6 |  |  |
| $75,000-99,999 | 13.4 |  |  |
| $100,000+ | 31.1 |  |  |
| Food insecurity |  | 53,058 | 92 (0.2%) |
| Food secure | 86.2 |  |  |
| Food insecure | 13.8 |  |  |
| Employment status past 1-2 weeks |  | 51,743 | 1,407 (2.6%) |
| Employed | 62.8 |  |  |
| Not employed | 37.2 |  |  |
| Health insurance |  | 52,964 | 186 (0.3%) |
| Private | 63.9 |  |  |
| None | 11.3 |  |  |
| Public | 24.8 |  |  |
| Chronic pain |  | 52,156 | 994 (1.9%) |
| Never or some days | 78.8 |  |  |
| Most or every day | 21.2 |  |  |
| High-impact chronic pain |  | 48,449 | 4,701 (8.8%)^1^ |
| Never or some days | 91.8 |  |  |
| Most or every day | 8.2 |  |  |

Weighted percent
NHIS = National Health Interview Survey; GED = general educational development diploma
^1^ As noted in the Methods section, this variable was not collected in the second quarter of 2020. The missingness is thus non-informative and affects only the precision of the estimates.

| Supplemental Table S2. Average predicted probabilities of prescription opioid use from fully adjusted models | | | | | | |
| --- | --- | --- | --- | --- | --- | --- |
|  | All adults | | With CP | | With HICP | |
| Survey year | % | 95% CI | % | 95% CI | % | 95% CI |
| 2019 | 12.6 | (12.1, 13.0) | 31.4 | (30.1, 32.7) | 43.0 | (40.8, 45.1) |
| 2020 | 11.4 | (10.8, 12.0) | 27.4 | (25.7, 29.1) | 39.9 | (36.6, 43.3) |
| Region of residence |  |  |  |  |  |  |
| Northeast | 9.3 | (8.5, 10.2) | 23.5 | (21.0, 26.0) | 32.8 | (28.5, 37.1) |
| North Central and Midwest | 11.9 | (11.1, 12.7) | 29.6 | (27.6, 31.6) | 44.3 | (40.3, 48.3) |
| South | 13.0 | (12.3, 13.8) | 31.9 | (30.0, 33.8) | 44.4 | (41.0, 47.9) |
| West | 12.2 | (11.4, 13.0) | 28.9 | (26.2, 31.6) | 40.4 | (36.2, 44.6) |
| Rural/urban county classification |  |  |  |  |  |  |
| Large central metropolitan | 11.7 | (11.0, 12.5) | 29.9 | (27.4, 32.5) | 42.7 | (38.6, 46.8) |
| Large fringe metropolitan | 11.8 | (11.1, 12.5) | 29.9 | (27.7, 32.0) | 39.3 | (35.2, 43.3) |
| Medium and small metro | 12.0 | (11.3, 12.7) | 28.7 | (26.8, 30.5) | 41.5 | (38.3, 44.8) |
| Nonmetropolitan | 12.4 | (11.3, 13.5) | 29.3 | (26.9, 31.7) | 43.0 | (38.1, 47.8) |
| Age |  |  |  |  |  |  |
| 18-44 | 10.8 | (10.1, 11.4) | 28.2 | (25.5, 30.8) | 38.2 | (33.3, 43.0) |
| 45-64 | 14.1 | (13.4, 14.8) | 33.1 | (31.3, 34.9) | 45.8 | (42.8, 48.8) |
| 65+ | 11.2 | (10.4, 11.9) | 26.0 | (24.2, 27.8) | 38.2 | (35.1, 41.4) |
| Female |  |  |  |  |  |  |
| Male | 10.6 | (10.1, 11.2) | 26.9 | (25.3, 28.4) | 39.4 | (36.7, 42.2) |
| Female | 13.1 | (12.5, 13.6) | 31.3 | (29.8, 32.8) | 43.2 | (40.5, 45.9) |
| Race/ethnicity |  |  |  |  |  |  |
| White | 12.7 | (12.2, 13.2) | 29.7 | (28.5, 30.9) | 42.5 | (40.2, 44.8) |
| Black | 11.7 | (10.5, 12.8) | 28.4 | (25.0, 31.8) | 37.7 | (32.1, 43.4) |
| Hispanic | 10.0 | (9.0, 11.1) | 28.8 | (24.9, 32.7) | 42.3 | (36.2, 48.4) |
| Other | 9.4 | (7.8, 11.0) | 27.8 | (22.1, 33.5) | 38.8 | (28.1, 49.6) |
| Immigrant status |  |  |  |  |  |  |
| US-born | 12.6 | (12.2, 13.1) | 29.9 | (28.7, 31.1) | 42.8 | (40.5, 45.0) |
| Foreign-born | 8.4 | (7.5, 9.3) | 24.8 | (21.3, 28.2) | 33.6 | (27.5, 39.8) |
| Marital status |  |  |  |  |  |  |
| Married | 12.6 | (12.0, 13.2) | 29.4 | (27.8, 31.1) | 43.9 | (40.8, 47.0) |
| Previously married | 13.6 | (12.8, 14.4) | 31.2 | (29.4, 33.0) | 40.7 | (37.5, 43.9) |
| Never married | 9.2 | (8.5, 10.0) | 25.9 | (23.0, 28.7) | 37.3 | (32.1, 42.6) |
| Educational attainment |  |  |  |  |  |  |
| Less than high school | 12.3 | (11.0, 13.7) | 31.0 | (27.6, 34.5) | 41.5 | (36.4, 46.6) |
| GED | 15.6 | (13.3, 17.9) | 33.9 | (29.0, 38.8) | 46.9 | (39.0, 54.8) |
| High school diploma | 11.3 | (10.5, 12.1) | 28.2 | (26.0, 30.4) | 40.1 | (36.2, 44.1) |
| Some college, no diploma | 13.4 | (12.5, 14.2) | 30.5 | (28.2, 32.8) | 43.9 | (39.8, 48.0) |
| Associate degree | 12.3 | (11.4, 13.2) | 30.3 | (27.6, 33.0) | 43.4 | (38.6, 48.1) |
| Bachelor’s degree | 11.1 | (10.3, 11.9) | 26.7 | (24.2, 29.3) | 37.6 | (32.5, 42.6) |
| Master’s or higher degree | 10.6 | (9.7, 11.5) | 27.0 | (23.7, 30.3) | 40.2 | (33.1, 47.3) |
| Family income |  |  |  |  |  |  |
| $0-34,999 | 12.1 | (11.4, 12.8) | 28.8 | (26.9, 30.6) | 40.5 | (37.6, 43.5) |
| $35,000-49,999 | 11.9 | (10.9, 12.9) | 29.2 | (26.3, 32.1) | 41.0 | (35.9, 46.0) |
| $50,000 - $74,999 | 12.2 | (11.3, 13.1) | 29.9 | (27.2, 32.6) | 44.0 | (39.2, 48.7) |
| $75,000-99,999 | 11.7 | (10.7, 12.8) | 28.2 | (24.8, 31.6) | 40.5 | (33.9, 47.2) |
| $100,000+ | 11.8 | (11.0, 12.7) | 30.7 | (27.6, 33.9) | 43.8 | (37.2, 50.4) |
| Food insecurity |  |  |  |  |  |  |
| Food secure | 11.1 | (10.7, 11.4) | 28.2 | (27.0, 29.4) | 39.8 | (37.4, 42.2) |
| Food insecure | 17.2 | (15.9, 18.4) | 33.0 | (30.3, 35.7) | 45.9 | (42.0, 49.8) |
| Employment status past 1-2 weeks |  |  |  |  |  |  |
| Employed | 9.8 | (9.4, 10.3) | 23.3 | (21.7, 25.0) | 37.1 | (33.0, 41.1) |
| Not employed | 15.0 | (14.3, 15.7) | 34.2 | (32.5, 35.9) | 43.5 | (41.1, 45.9) |
| Health insurance |  |  |  |  |  |  |
| Private | 11.7 | (11.2, 12.2) | 29.8 | (28.3, 31.3) | 44.8 | (41.8, 47.9) |
| None | 7.7 | (6.6, 8.8) | 19.4 | (15.5, 23.3) | 24.1 | (17.1, 31.2) |
| Public | 13.9 | (13.1, 14.7) | 30.9 | (29.0, 32.7) | 42.1 | (39.3, 44.9) |
| N | 50,773 | | 11,773 | | 4,298 | |

CP = chronic pain; HICP = high-impact chronic pain; GED = General educational development diploma

Prevalence estimate and their 95% confidence intervals. Estimation adjusts for NHIS complex sampling design.

All covariates entered into the models simultaneously.
