# Supplementary material for: Prevalence and correlates of prescription opioid use among US adults, 2019–2020
Source: PLoS One. 2023 Mar 2;18(3):e0282536. doi: 10.1371/journal.pone.0282536 (PMC9980762; doi:10.1371/journal.pone.0282536)
Supplement: Attachment — Submitted filename: PAIN-OPI-NHIS R1 RM 23-02-03.docx [file pone.0282536.s002.docx]

Journal: PLOS One

Title: Prevalence and Correlates of Prescription Opioid Use among US Adults in 2019-2020.

ID: PONE-D-22-25884 (first revision)

Authors: Anna Zajacova; Hanna Grol-Prokopczyk; Merita Limani, Christopher Schwarz, Ian Gilron.

February 3, 2023

Dear Dr. Latham-Mintus and anonymous reviewers,

Thank you for considering our work for possible publication in *PLOS One* and the opportunity to revise and strengthen our manuscript.

We greatly appreciate the positive assessment from both reviewers, as well as their constructive and insightful suggestions.

We addressed all comments and revised the manuscript accordingly. In addition, we made further changes to our writing and analysis:

- We added relevant new references
- We have heavily revised the text for clarity, adding information and richer interpretation of findings
- We redid Figure 2. The figure originally showed coefficient estimates but we converted it to showing exponentiated coefficients, that is, prevalence ratios, which are a better, more intuitive, metric
- We changed our specification of the age covariate from a continuous quadratic specification to a three-level categorical, which simplifies how this covariate is interpreted.
- We switched Supplemental Table 2 with Table 2 because we realized we used material from the latter source in writing up our results. This switch better aligns the presentations of our findings across the text and tables.

We emphasize that all our conclusions and even individual estimates remain unchanged from the original version.

We hope the current version is found suitable for publication in *PLOS One*.

**Reviewer #1**

This is an interesting paper which describes the prevalence of opioid use in the USA and factors associated to opioid use. The study is well conducted and its results well presented, although some improvements in Methods are necessary.

Thank you for your positive assessment of our analysis, as well as for your constructive comments. We made all the changes you suggested, including in the Methods section where we expanded the “Statistical analyses” section.

Abstract
Please define the acronyms at their first appearance and use them through the abstract.

Thank you. Previously we failed to define “the US;” we have removed the acronym from the abstract altogether. All other acronyms and abbreviations are defined the first time they appear and then are used consistently.

Insert some more quantitative results.

We revised the abstract and added select estimates, both prevalences in the total population and prevalence ratios describing differences or patterns across geographic and individual characteristics.

Introduction
Please define the acronyms at their first appearance and use them through the text.

Thanks for noticing. We defined all acronyms and abbreviations at first use, such as NHIS, US, SES, etc. We kept the acronym STROBE, which stands for “The Strengthening the Reporting of Observational Studies in Epidemiology”, because a thorough search of the literature shows this acronym is used without a full definition given its widespread use. We are happy to add the full definition if the reviewer prefers.

We wish to note that there is one place in the manuscript we perhaps appear not to define an acronym: in the Data subsection of the Methods section, where we use the name IPUMS without defining it. This is, however, not an omission; it is the correct usage. IPUMS, which historically stood for Integrated Public Use Microdata Series, in 2016 stopped treating its name as an acronym. Or as IPUMS’ own [Mission and Purpose](https://www.ipums.org/mission-purpose) source states: “IPUMS originally stood for Integrated Public Use Microdata Series. In 2016, all of our data projects took on the IPUMS prefix as part of their project name (e.g., IPUMS NHGIS, IPUMS Terra, etc.). Since not all these projects are microdata and some have access conditions that limit their usage, it became inaccurate to describe IPUMS as a "public use” microdata series. Since then, we've just gone by IPUMS, and no longer treat it as an acronym.”

Please delete the first sentence “This analysis presents a comprehensive portrait of prescription opioid use (POU) among US adults in 2019/2020.”
We followed this suggestion and deleted the first sentence.

Methods
Please move the first sentence at the end of the Data section.

We moved the sentence; it fits better at the end of the Data section indeed.

Please include the sources mentioned in the sentences “are available here for 2019 and here for 2020.” and “Full details about the prompt are available on page 287 here.” among the References.
We made this change. The text now includes formal references for these three sources rather than the in-text links.

Please move the sentence “All variables are self-reported” at the end of the Variables section.

We moved this sentence as you suggested.

Please remove the variable names in the Variables section.

We removed the variable names.

Please delete this sentence “We coded the outcome as 1 if 88 respondents reported taking prescription opioids in the past 12 months and 0 otherwise.”

We removed this sentence; we agreed it wasn’t necessary.

Please explain what the authors mean by food insecurity.

Yes, that’s a good point! We have substantially expanded our description of this variable, including adding a reference to the original guide from the US Department of Agriculture with a definition of food security. The text in the methods section now states: “Food insecurity indicator is based on a ten-question scale, administered by NHIS following the US Department of Agriculture Guide, that captures a family’s “access by all people at all times to enough food for an active, healthy life" (18, p.6) by asking questions such as whether anyone in the family ate less or skipped meals because of not enough money, or ever went hungry, or whether family couldn’t afford to eat balanced meals, all questions with respect to prior month. We dichotomized this scale as 1 for a positive response to any food insecurity items versus 0 for food-secure.”

Please rename the section Approach into Statistical analyses.

Done.

The paragraph from line 114 to line 124 should be moved in the Results section. Please add a few more details on the Statistical Methods.

The text in lines 114-124 explained the methods (that is, the analytic approach). We revised the text heavily and expanded it as you requested. That’s an important improvement, thanks for the suggestion. The revised text is now clearly the “statistical approach” rather than a description of results; thus, we have retained it under the Methods heading. We hope this revised text fits the section and that it includes adequate detail for readers to know precisely how we analyzed the data.

Please add the source “NHIS IPUMS guidelines” in the References.

We added the source of this citation into the References.

Figure 2
Please check the data for food-insecure vs secure. The results for all individuals are very different from those of the two subgroups.

Great observation! The results are correct, and we are thrilled you noticed this unusual pattern because, actually, this is one of the major strengths of our study.

You noticed that in the model for all adults, food insecurity is associated with **55%** higher prevalence of POU (PR=1.55, 95% CI 1.43, 1.68) than the prevalence among food-secure respondents, net of all covariates. Among adults with chronic pain and high-impact chronic pain, it’s ‘only’ **17%** and **15%** higher prevalence, respectively (PR=1.17, 95% CI 1.06, 1.29 in the CP model, and PR=1.15, 95% CI 1.04, 1.29 in the HICP).

What this implies is that much of the apparent high opioid use among food-insecure adults (net of all other covariates) is due to their high pain prevalence; if we only look at people with chronic pain, the food-insecure still have higher POU than the food-secure adults, but the difference isn’t as dramatic.

Indeed, we can look more into the data: chronic pain occurs among 19.1% of food-secure adults, but nearly twice that, 34.4%, of food-insecure adults. High-impact chronic pain prevalence difference is even more stark: 6.5% of the food-secure but 18.5% among the food-insecure.

We have revised the text of the Results section subtly to highlight this strength of the analysis by mentioning the estimates for select covariates in the general population and among those with pain.

Moreover, we also added text in the Discussion section, specifically noting as a strength of our study that the estimates for the three subsamples allow readers to see what disparities are largely due to pain prevalence differences versus differences in opioid use even conditioning on pain or high-impact chronic pain.

Table 2 and Supplementary Tables 1 and 2
Please provide the acronym definition in the footnotes.

We have added acronym definitions to the Tables, as well as Supplemental Tables.

**Reviewer #2**

The article is well written and acknowledges the problems with this type of database--self-reporting for example.

Thank you for your positive assessment of our analysis.

Please define the term "pain patient" on line 51, especially in light of the later classifications of individuals with pain and individuals with high impact pain.

Thank you for noticing. We deleted the phrase “pain patient” from the manuscript. We revise the phrase to “adults with pain,” which is precise and correct, as we are not trying to make any statements about the patient social role but we simply capture that the respondents reported having chronic pain or high-impact chronic pain.

The one place in the document, where we specifically mention prior research on adults who are treated for pain, also uses this less parsimonious but more accurate term. We write “Third, nearly all research has focused either on the general population or adults who are treated for pain, but not both. Adults who receive treatment for pain are a select group, since many adults with pain do not receive treatment for pain.”
